# Comparison of adaptive optical scanning holography based on new evaluation methods

**DOI:** 10.1038/s41598-023-46851-0

**Published:** 2023-11-11

**Authors:** Jilu Duan, Yaping Zhang, Yongwei Yao, Qingyang Fu, Bing Zhang, P. W. M. Tsang, Ting-Chung Poon

**Affiliations:** 1 Yunnan Provincial Key Laboratory of Modern Information Optics (LMIO), Kunming University of Science and Technology, Kunming, 650500 Yunnan China; 2 Department of Electronic Engineering, City University of Hong Kong, Hong Kong, China; 3 Bradley Department of Electrical and Computer Engineering, Virginia Tech, Blacksburg, VI 24061 USA

**Keywords:** Imaging and sensing, Displays

## Abstract

Adaptive Optical Scanning Holography (AOSH) represents a powerful technique that employs an adaptive approach to selectively omit certain lines within holograms, guided by the utilization of Normalized-Mean-Error (NME) as a predictive measure. This approach effectively diminishes scanning time and conserves the storage space required for data preservation. However, there exists alternative methods superior to NME in terms of evaluating the model’s efficacy. This paper introduces two novel methods, namely Normalized-Root-Mean-Square-Error (NRMSE) and Normalized-Mean-Square-Error (NMSE), into the AOSH system, leading to the development of NRMSE-AOSH and NMSE-AOSH. These new systems aim to further minimize duration of holographic recording. Through a comparative analysis of hologram lines between the two newly proposed AOSH systems and the original AOSH, we demonstrate that both NRMSE-AOSH and NMSE-AOSH effectively reduce the number of hologram lines while maintaining the hologram’s informational content. Among the three methods, our two new methods exhibit better performance compared with the original method.

## Introduction

Digital holography, a well-established technique, utilizes charge-coupled devices (CCDs) in place of conventional recording materials to capture interference fringes, thereby enabling the digital recording of three-dimensional object scenes^1,2^. While digital holography offers numerous advantages over its traditional counterpart, its resolution and field of view are constrained by the size of the CCD and its pixel size. To address these limitations, researchers have pursued various approaches, such as interpolation and iteration techniques to enhance the resolution of digital hologram reconstruction^3^, as well as digital holography based on synthetic aperture technology^4^.

Another solution, known as Optical Scanning Holography (OSH)^5–7^, was initially proposed by Poon and Korpel in the 1970s^5^. OSH, a distinct form of digital holography, involves the projection of a time-dependent Fresnel zone plate (TDFZP)^7^ onto an object, with the scattered light at each point of the object captured by a photo-detector. By raster scanning a 3-D object with the TDFZP, holographic data can be obtained for the object. OSH-based holograms are limited only by the scanning range, allowing the acquisition of large-scale digital holograms. So far, The OSH technology has been applied in various areas, such as fluorescence microscopy^8,9^, remote sensing^10^, and three-dimensional (3-D) imaging recognition^11,12^. In recent developments, off-axis OSH systems have been proposed to reduce the complex electronic devices and their effectiveness have been verified by experiments^13–15^. However, the mechanical progressive scanning required for hologram acquisition leads to prolonged scanning times and large data storage requirements if the size of the object is large. To mitigate these challenges, researchers have sought to minimize the number of scanning points or reduce data storage through encoding, while preserving essential information .

Tsang et al. proposed Compressive Optical Scanning Holography (COSH)^16^ and Adaptive Optical Scanning Holography (AOSH)^17^. COSH and AOSH effectively reduce spatial redundancy in holograms through predictive coding compression and adaptive error modeling, respectively. Liu et al. combined horizontal parallax hologram (HPO) with OSH to obtain vertical bandwidth-limited hologram (VBLH)^18^, addressing the under-sampling issue by utilizing horizontal low-pass filtering while reducing the sampling of hologram lines. Among these approaches, AOSH represents a special solution that employs the Normalized-Mean-Error (NME) method to adaptively reduce hologram scanned lines. NME is an error evaluation method normalized by Mean-Absolute-Error (MAE) and is suitable for describing uniformly distributed errors. However, various error evaluation methods exist with distinct assessment performance. Mean-Square-Error (MSE) and Root-Mean-Square-Error (RMSE) are two commonly used evaluation methods in the analysis of experimental results. In this paper, we aim to demonstrate the performance of AOSH using these two new methods, and compare with the original NME approach. Following this introduction, we present the experimental methodology in Section 2. Section 3 showcases the experimental results and evaluations, comparing the three AOSH approaches using different error evaluation methods. We highlight and compare the results obtained from the three AOSH methods. Finally, Section 4 provides discussions summarizing the findings of the study.

## Methods

### Overall view of the OSH system and AOSH system

**Figure 1:**
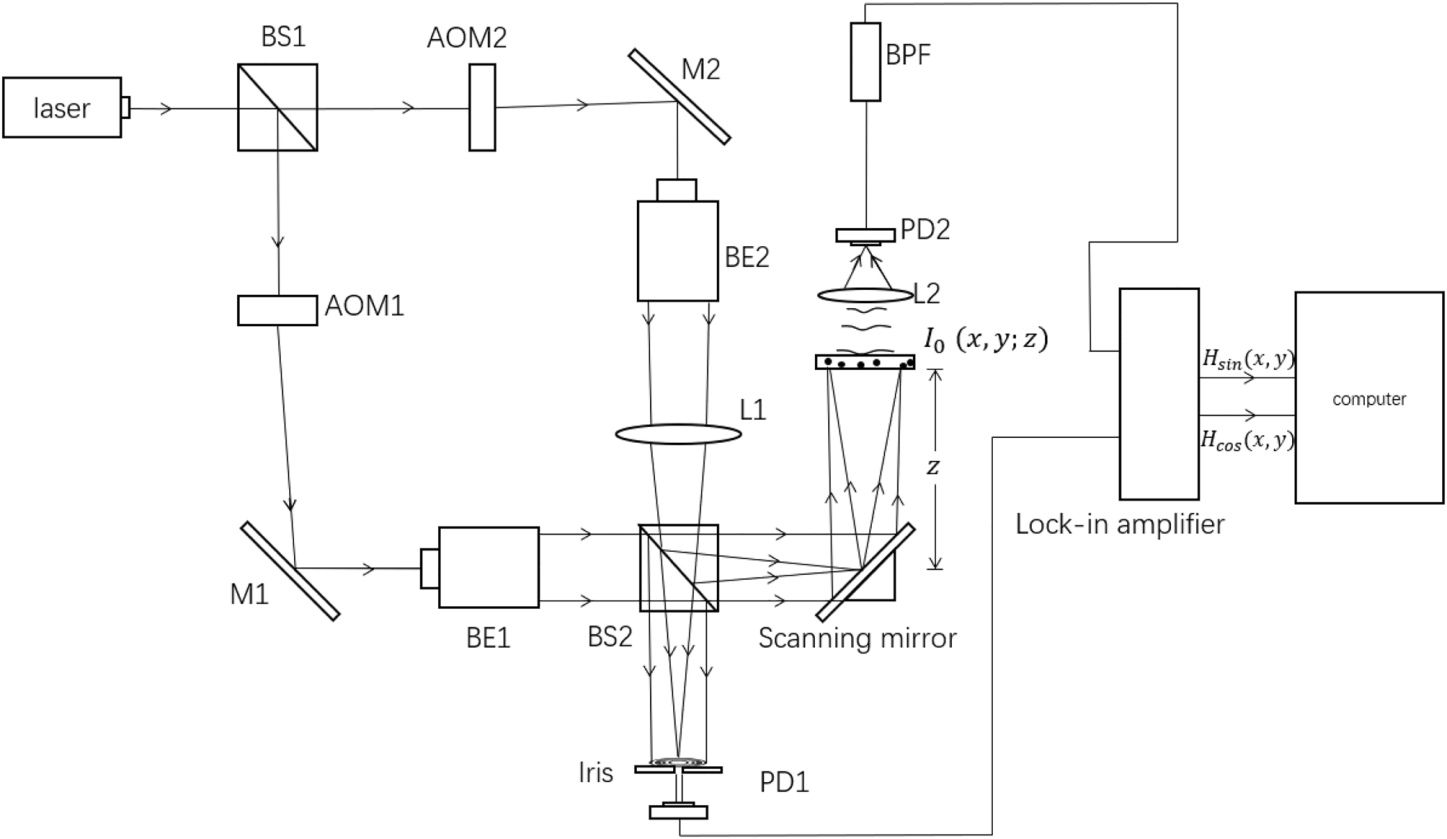
The setup of OSH to record the hologram of object $I_0 (x,y;z)$. *BS1,2* beam splitter, *AOM1,2* acoustic-optic modulator, *M1,2* mirrors, *BE1,2* Beam expander, * L1,2* lens, *PD1,2* photodetector.

Prior to introducing the AOSH system, it is necessary to provide a concise overview of the OSH system. As a comprehensive explanation of OSH can be found in numerous existing literature^6,16,17^, only a succinct elucidation of the OSH principle will be presented. The experimental configuration of OSH is depicted in Fig. 1. The emitted laser of wavelength $\lambda =532 \, {\text{nm}}$ is split into two by beam splitter BS1. The temporal frequencies of two beams are modulated into $$ {\omega _0}\mathrm{{ + }}\Omega \ $$ and ${\omega _0}$ by acoustic-optic modulator1 (AOM1) and acoustic-optic modulator2 (AOM2) , respectively. Thus, the heterodyne frequency ${\Omega }$ between these two beams is introduced. The upper beam is first collimated by beam expander2 (BE2) and then provides a spherical wave on object $I_0 (x,y;z)$ through the focusing action by lens1 (L1). The other beam is collimated by beam expander1 (BE1); hence a plane wave is projected onto the object. The spherical wave and plane wave are combined by beam splitter2 (BS2), generating a heterodyne interference pattern on the scanning mirror. The interference pattern is known as a time-dependent Fresnel zone plate (TD-FZP)^7^. The TD-FZP oscillates at $\Omega$. The scanning of the object is done by the scanning mirror, which can scan the 3D object uniformly in a row-by-row manner. The scattered light transmitted through the object is converged to photodetector1 (PD1) by lens2 (L2). The PD1 collects the light and sends the electrical signal containing the holographic information of the scanned object to the bandpass filter (BPF) which tunes to the electrical signal at frequency $\Omega$. Next, the signal from BPF goes into the lock-in amplifier. In the meantime, photodetector2 (PD2) delivers a heterodyne signal into a Lock-in amplifier as a reference signal. Finally, a complex hologram can be obtained by combining the in-phase output and the quadrature output of the lock-in amplifier. The in-phase and the quadrature-phase outputs of the lock-in amplifier give a sine hologram ${H_{\sin }}(x,y)$, and cosine hologram ${H_{\cos }}(x,y)$ as follows^7^:

1 $$\begin{aligned}{} & {} {H_{\sin }}(x,y) = \int {{I_0}(x,y;z) * \frac{1}{{\lambda z}}\sin [\frac{\pi }{{\lambda z}}({x^2} + {y^2})]dz},{} & {} \end{aligned}$$

and

2 $$\begin{aligned}{} & {} {H_{\cos }}(x,y) = \int {{I_0}(x,y;z) * \frac{1}{{\lambda z}}\cos [\frac{\pi }{{\lambda z}}({x^2} + {y^2})]dz}.{} & {} \end{aligned}$$

where $I_0 (x,y;z)$ denotes the intensity distribution of the 3-D object, $\lambda$ is the wavelength of light in free space, and * denotes 2-D convolution involving *x* and *y*^6^.

According to Eqs. (1) and (2), the resulting complex hologram *H*(*x*, *y*) in the computer can be expressed as

3 $$\begin{aligned}{} & {} \begin{aligned} H(x,y)&={H_{\cos }}(x,y) + i{H_{\sin }}(x,y)\\&=\int {{I_0}(x,y;z) * \frac{1}{{\lambda z}}\exp [i\frac{\pi }{{\lambda z}}({x^2} + {y^2})]dz}. \end{aligned}{} & {} \end{aligned}$$

**Figure 2:**
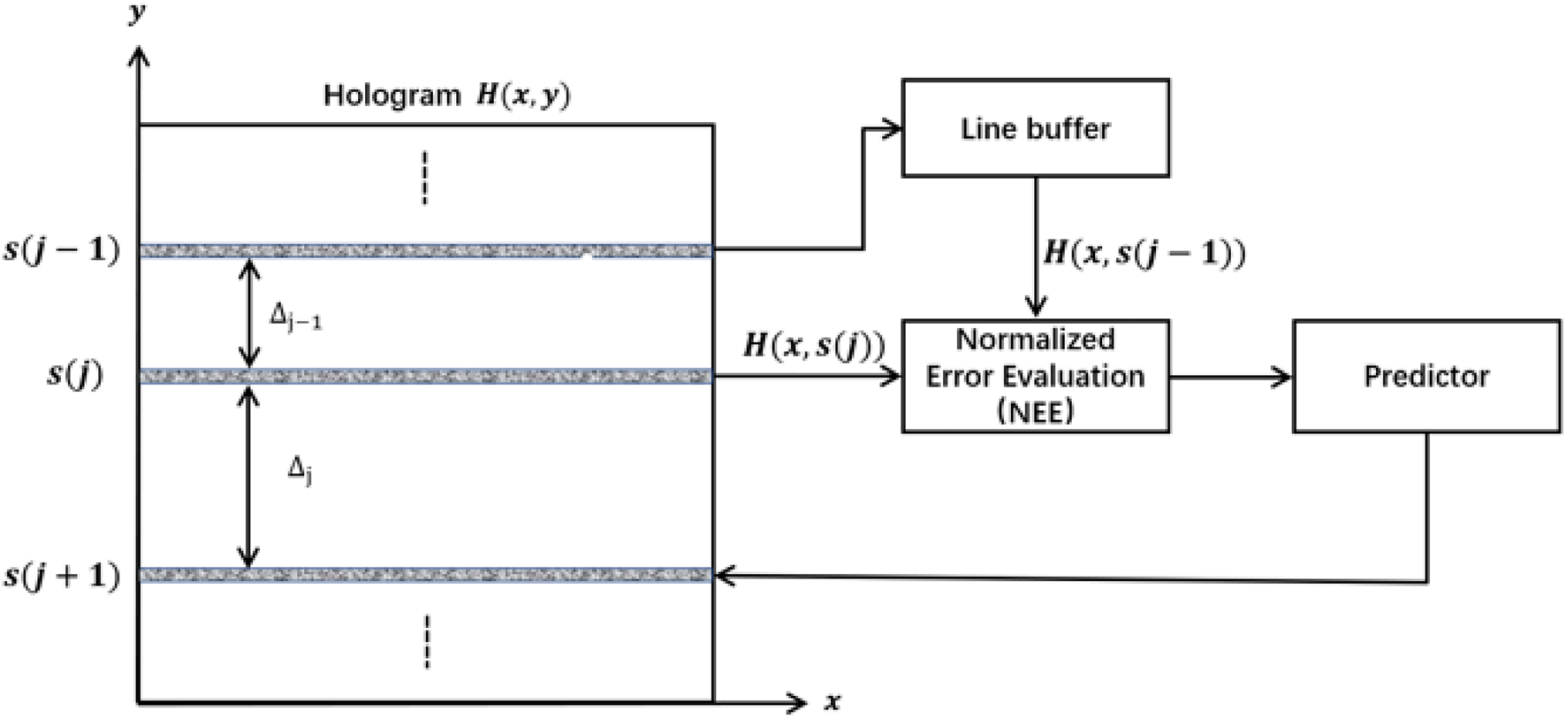
Concept of the AOSH scanning mechanism^17^.

In the OSH configuration, the hologram pixels are obtained sequentially in a line-by-line manner from the lock-in amplifier, synchronized with the movement of the TD-FZP. The AOSH method introduces a selection mechanism for the hologram lines, employing an error evaluation approach to estimate the level of “smoothness” between a pair of hologram lines. This evaluation facilitates the identification and omission of redundant information within the hologram lines. The scanning mechanism of AOSH is illustrated in Fig. 2. The key aspect of the technique lies in the fact that AOSH adjusts the gap between hologram lines by calculating the error evaluation.

The concept of AOSH is explained as follows. We denote the position of rows that will be scanned with the sequence $S = s{(j)_{0 \le j < r}}$, where *j* is the index of *S*, *s*(*j*) is the position of the *j*th scan row, and *r* is the total scan rows. The expression of the hologram line located in *s*(*j*) is *H*(*x*, *s*(*j*)) , and its previous row and next row is $H(x,s(j-1))$, and $H(x,s(j+1))$, respectively. The separation between the hologram line at *s*(*j*) and the hologram line at $s(j-1)$ and $s(j+1)$ is denoted as ${\Delta _{j - 1}}$ and ${\Delta _{j}}$, respectively. Initially, *H*(*x*, *s*(0)) and *H*(*x*, *s*(1)) need to be acquired. Suppose the current scanning hologram line is *H*(*x*, *s*(*j*)), and the previous hologram line is $H(x,s(j-1))$ stored in the buffer. In AOSH, We calculate the error evaluation between the $H(x,s(j-1))$ and *H*(*x*, *s*(*j*)), which can reflect the “smoothness” of a pair of hologram lines, to predict the position of the next scan row $s(j+1)$. The predictor estimates ${\Delta _{j}}$, which is the most significant aspect of AOSH, always need the error evaluation to measure the similarity between the previous hologram line and the current hologram line. As the error evaluation between $H(x,s(j-1))$ and *H*(*x*, *s*(*j*)) is smaller, the gap between *s*(*j*) and $s(j+1)$ becomes wider. The separation between the current and the next scan row is decided by the Predictor as

4 $$\begin{aligned}{} & {} {\Delta _j} = (1 - NE{E_j}) \times {\Delta _s} + {\Delta _{MIN}},{} & {} \end{aligned}$$

where $NE{E_j}$ denotes the normalized error evaluation of *j*th scan row, which range is 0 to 1, ${\Delta _{s}}$ and ${\Delta _{MIN}}$ are the factors which decide the scanning speed and the hologram quality. The next position of scan row is expressed as

5 $$\begin{aligned}{} & {} s(j + 1) = s(j) + {\Delta _j}.{} & {} \end{aligned}$$

**Figure 3:**
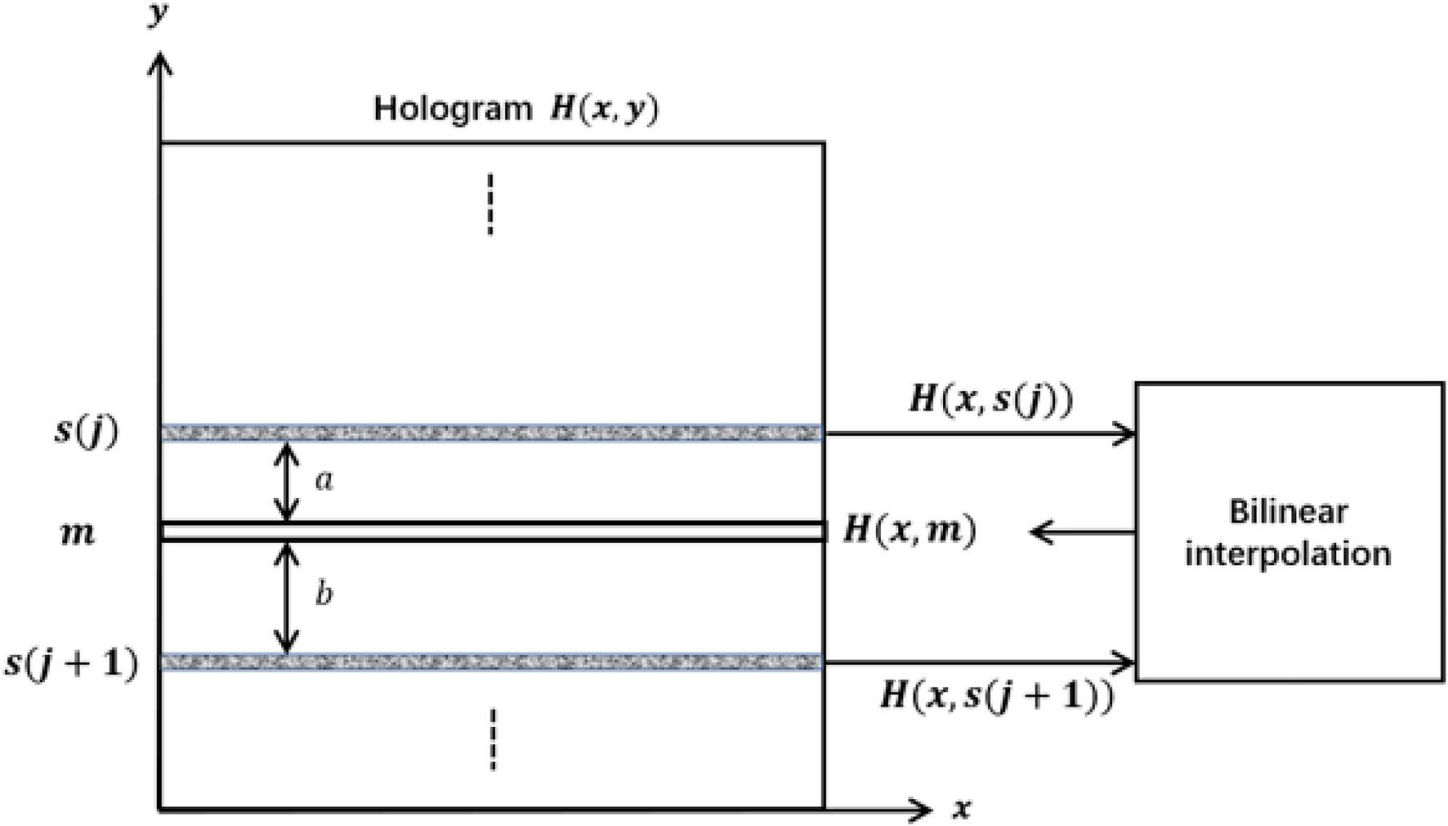
Filling a row of pixels between a pair of hologram lines.

It can be seen that a small $NE{E_j}$ results in large $\Delta _j$, and the separation between the current scan row and the next scan row becomes wider. The steps are iteratively performed until the final row of the object scene has been scanned. After capturing all the hologram lines, the regions between adjacent hologram lines are filled with bi-linear interpolation as shown in Fig. 3. Given 2 adjacent hologram lines *H*(*x*, *s*(*j*)) and $H(x,s(j+1))$, the missing hologram line *H*(*x*, *m*) at vertical position ‘*m*’ (where $s(j)< m < s(j + 1)$) between them is determined as

6 $$\begin{aligned}{} & {} H(x,m) = [\frac{b}{{a + b}}]\times H(x,s(j)) + [\frac{a}{{a + b}}]\times H(x,s(j + 1)),{} & {} \end{aligned}$$

where *a* and *b* denote the vertical distance between *H*(*x*, *m*) and *H*(*x*, *s*(*j*)) and *H*(*x*, *m*) and $H(x,s(j+1))$, respectively. The term $\frac{b}{{a + b}}$ and $\frac{a}{{a + b}}$ are the weight factors, which represent the contribution of *H*(*x*, *s*(*j*)) and $H(x,s(j+1))$ to *H*(*x*, *m*), respectively.

### NRMSE and NMSE in AOSH

In the preceding section, we have discussed the utilization of normalized error evaluation (NEE) in AOSH, as depicted in Fig. 2. It is worth noting that the reference 17 introduces AOSH for the first time^17^ by employing Normalized-Mean-Error (NME) as a specific NEE method. NME is the normalized Mean-Absolute-Error(MAE) and owns the same characteristics as MAE. Nevertheless, it has been suggested that Root-Mean-Square-Error (RMSE) exhibits superior performance to MAE in the evaluation of models. MAE is affected by a large number of average error values, and cannot fully reflect some large errors compared with RMSE^19^. Considering this, we hypothesize that normalized Root-Mean-Square Error (NRMSE) may exhibit superior performance within the AOSH framework. Additionally, Mean-Square Error (MSE) represents another commonly used error evaluation method, capturing the quadratic nature of errors and providing detailed insights into error analysis results. Furthermore, Normalized Mean-Square Error (NMSE) serves as a normalized variant of MSE. Both NRMSE and NMSE present alternative approaches to normalized error evaluation (NEE) within the AOSH context. In this section, we will introduce the specific formulations of these two error evaluation methods in AOSH and elucidate their significance. To facilitate clarity, we will refer NRMSE-based AOSH as NRMSE-AOSH, NMSE-based AOSH as NMSE-AOSH, and NME-based AOSH as NME-AOSH.

In the following equations, we denote *H*(*x*, *s*(*j*)) as the current hologram line; $H(x,s(j-1))$ is the previous hologram line which has been stored in the buffer. *x* represents the coordinate position of the pixel on the hologram line, and *X* is the total number of pixels on the hologram line. $NMS{E_j}$ and $NRMS{E_j}$ denote the NMSE and NRMSE between the $(j-1)^{th}$ and $(j)^{th}$ scan row.

The NMSE and NRMSE in AOSH can be respectively expressed as

7 $$\begin{aligned}{} & {} NMS{E_j}&= \frac{{\frac{1}{X}\sum \nolimits _{{x} = 0}^{X\mathrm{{ - }}1} {{{(H(x,s(j)) - H(x,s(j - 1)))}^2}} }}{{\frac{1}{X}\sum \nolimits _{{x} = 0}^{X\mathrm{{ - }}1} {{{(H(x,s(j)))}^2}} }}, \end{aligned}$$

8 $$\begin{aligned}{} & {} NRMS{E_j}&= \sqrt{NMS{E_j}} \mathrm{{ = }}\frac{{\sqrt{\frac{1}{X}\sum \nolimits _{{x} = 0}^{X\mathrm{{ - }}1} {{{(H(x,s(j)) - H(x,s(j - 1)))}^2}} } }}{{\sqrt{\frac{1}{X}\sum \nolimits _{{x} = 0}^{X\mathrm{{ - }}1} {{{(H(x,s(j)))}^2}} } }}, \end{aligned}$$

and the NME which is the original method in the AOSH can be expressed as^9^

9 $$\begin{aligned}{} & {} NM{E_j}\mathrm{{ = }}\frac{{\frac{1}{X}\sum \nolimits _{{x} = 0}^{X\mathrm{{ - }}1} {|H(x,s(j)) - H(x,s(j - 1))|} }}{{\frac{1}{X}\sum \nolimits _{{x} = 0}^{X\mathrm{{ - }}1} {|H(x,s(j))|} }}.{} & {} \end{aligned}$$

The NMSE and NRMSE ,which both are bounded within the range [0,1], compute the average difference between correspondence pixels between 2 consecutive rows of hologram pixels. However, the two methods do not evaluate the error in the same way. Because NMSE squares the difference between pairs of hologram lines, it will give more penalties to the errors between hologram lines. In other words, NMSE-AOSH is easier to observe the smoothness between holograms, which will help AOSH to skip some similar information more adaptively. For instance, for the same group of holographic line pairs, if a pair of hologram lines corresponding pixels change smoothly, where the average absolute error is less than one ($\frac{1}{X}\sum \nolimits _{\mathrm{{x}} = 0}^{X\mathrm{{ - }}1} {|H(x,s(j)) - H(x,s(j - 1))|} < 1$), the value of $NMS{E_j}$ is smaller than that of $NM{E_j}$ because the average absolute error is squared, which means that ${\Delta _{j}}$ calculated by NMSE is larger than that calculated by NME, as shown in Eq. (2). In other words, the smoothness between the hologram line pairs can be amplified by NMSE, and the AOSH scanned lines will be reduced. The NRMSE, derived from the square root of NMSE, shares a similar characteristic with NMSE and is adept at detecting subtle variations within the data. Therefore, NRMSE-AOSH can also provide an enhancement to the AOSH system.

## Results

In this section, we will assess and compare the performance of NMSE and NRMSE with the original method (NME) in AOSH. The experimental setup in Fig. 1 is outlined as follows. The wavelength of the laser beam is 532 nm, and the center frequency of AOM1 and AOM2 is 120 MHZ, providing a heterodyne signal of 10 kHz. To facilitate a comprehensive evaluation of these methods, we have employed three AOSH approaches to capture holograms of two objects: the United States Air Force resolution chart and a dice. The object United States Air Force resolution chart is a transmissive object and located at around 150 mm from the scanning mirrors. Its physical size is 20 mm × 20 mm. The dice is a reflection type object located at around 320 mm from the scanning mirrors. Its physical size is 25 mm × 25 mm. The classical OSH technique is utilized to record holograms of the two objects. The physical parameters in the experiment are outlined in Table 1.

**Table 1:** Parameters in the OSH/AOSH acquisition process.

Hologram pixel size	$12 \upmu {\text{m}} \times 12 \upmu {\text{m}}$
Hologram size	2048 × 2048
Wavelength of optical beam	${532 \text{nm}}$

**Figure 4:**
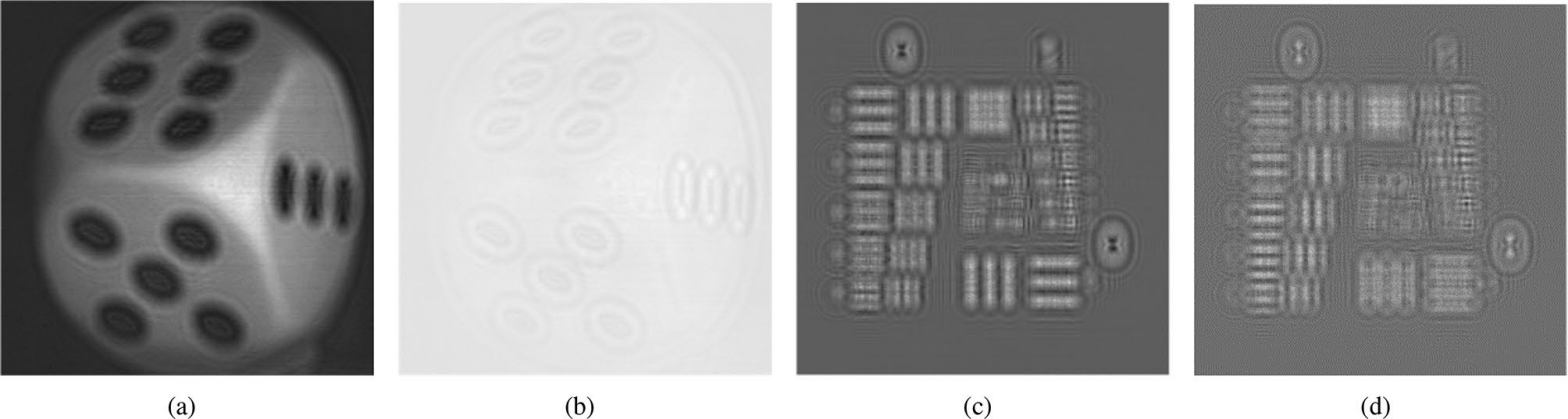
(**a**) Cosine hologram of the dice; (**b**) Sine hologram of the dice; (**c**) Cosine hologram of the United States Air Force resolution chart; (**d**) Sine hologram of the United States Air Force resolution chart.

**Figure 5:**
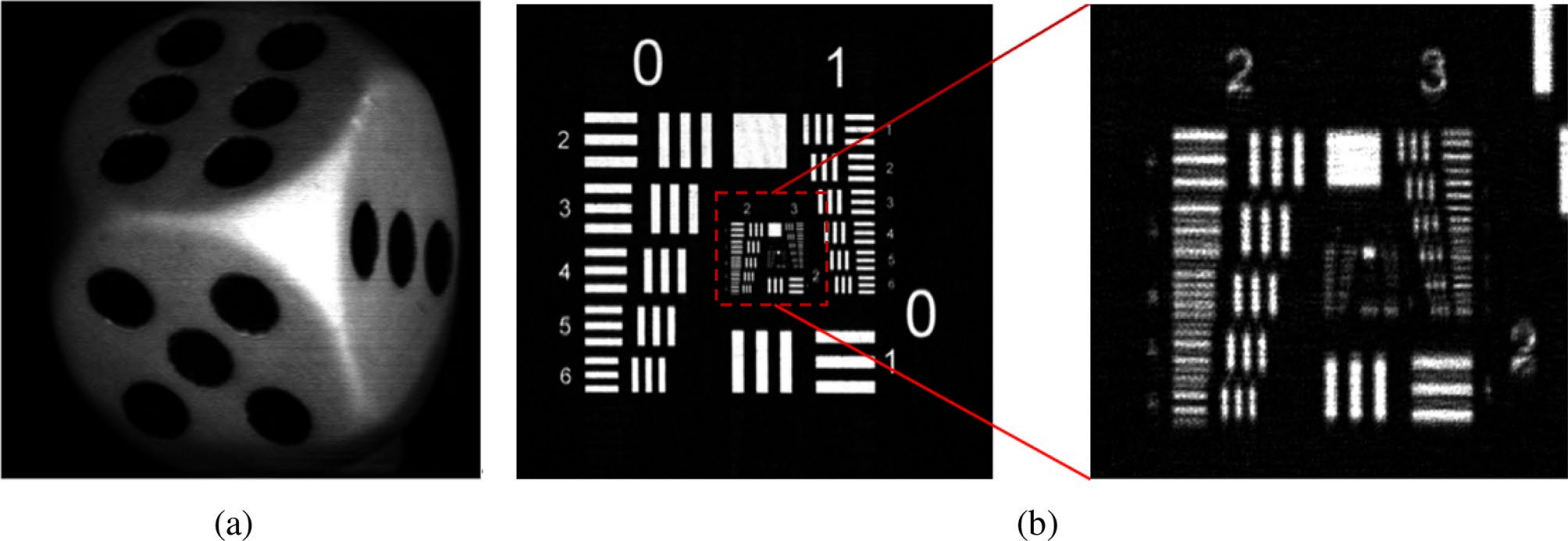
(**a**) Reconstructed image of the dice; (**b**) Reconstructed image of the United States Air Force resolution chart.

**Figure 6:**
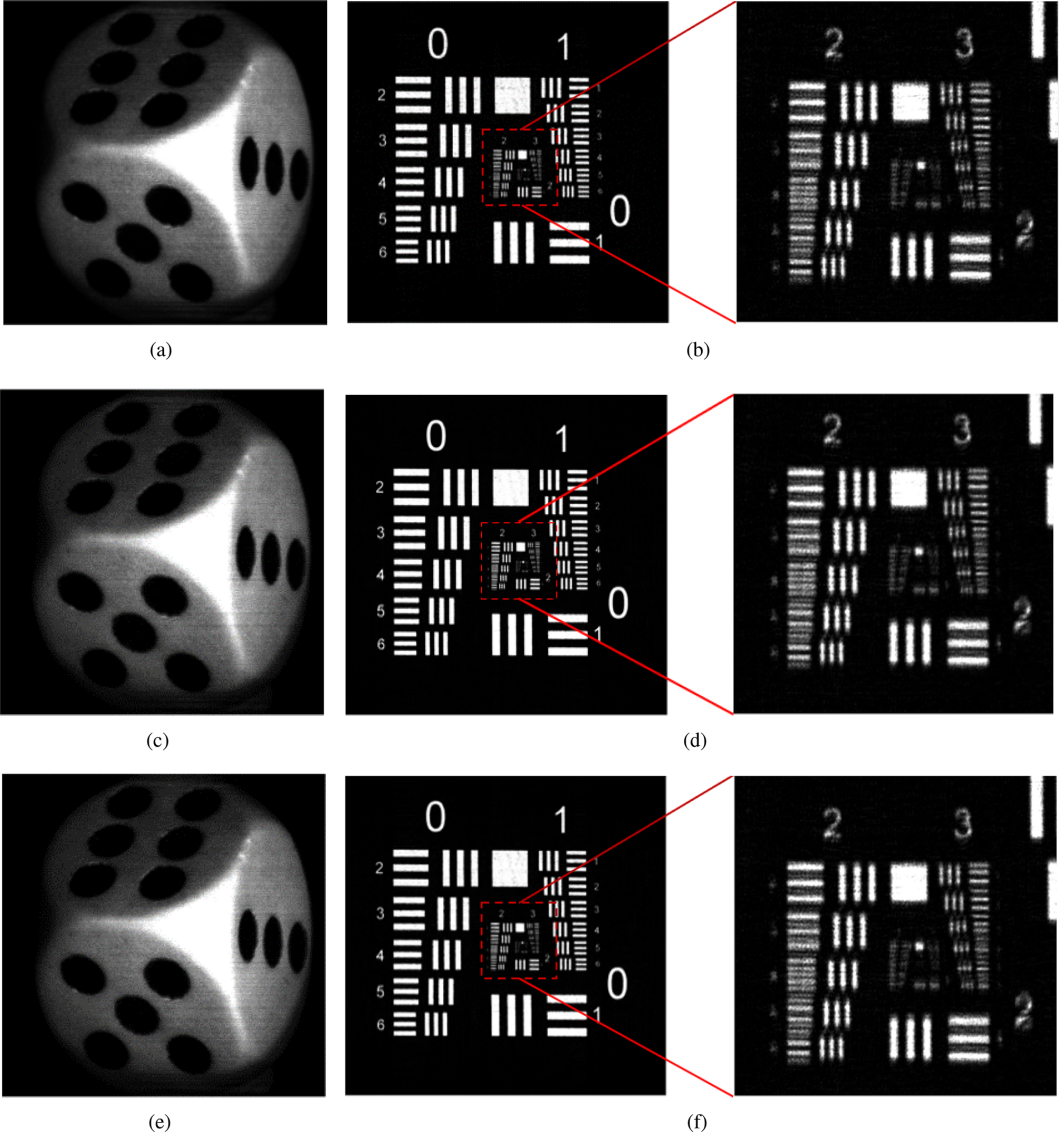
(**a**, **b**) Reconstructed image of the dice and the resolution chart with NRMSE-AOSH; (**c**, **d**) reconstructed image of the dice and the resolution chart with NMSE-AOSH; (**e**, **f**) reconstructed image of the dice and the resolution chart with original method (NME-AOSH).

The cosine and the sine holograms of the 2 objects, and their reconstructed images at the focused plane, are shown in Figs. 4a–d and 5a,b, respectively. Next, we apply AOSH method using NRMSE and NMSE respectively to capture the hologram of these two objects, based on ${\Delta _{MIN}} = 1$, ${\Delta _{s}}$ is changed from 2 to 7. In the case of a fixed ${\Delta _{MIN}}$, as the value of ${\Delta _{s}}$ increases in AOSH, the number of scanned lines decreases, leading to a decrease in the quality of the reconstructed image. On the other hand, the variability of ${\Delta _{s}}$ serves as an indicator of the robustness of NRMSE-AOSH and NMSE-AOSH in the context of AOSH. To assess and compare the performance of NRMSE-AOSH and NMSE-AOSH with the original method (NME-AOSH), we employ the NME-AOSH technique to capture holograms of the two objects under identical experimental conditions. The reconstructed images obtained from three different methods of AOSH are depicted in Fig. 6. It is evident that the reconstructed images achieved through NRMSE-AOSH and NMSE-AOSH (Fig. 6a–d) closely resemble the image obtained using the original approach (NME-AOSH) (Fig. 6e,f).

Apart from the visual inspection, we evaluate the difference in compression performance between the two new methods and AOSH based on the original method (NME-AOSH). The compression performance is evaluated by two aspects: the fidelity of the reconstructed image and the number of scanned lines for the hologram. The fidelity of the reconstructed images is measured in Peak-Signal-to-Noise-Ratio (PSNR) to compare with the reconstructed image of the holograms acquired with classical OSH.We then evaluate improvement of compression rate (scanned lines) between new methods (NRMSE-AOSH and NMSE-AOSH) and the original method (NME-AOSH) by evaluating the ratio of the difference between the number of scanned lines in original method and the two new methods to the number of scanned lines in the original method. The compression rate *R* is expressed as

10 $$\begin{aligned}{} & {} {R = }\frac{{line{s_{original}} - line{s_{new}}}}{{line{s_{original}}}} \times 100\% , \end{aligned}$$

For the dice and United States Air Force resolution chart, the compression performance of the two new methods (NRMSE-AOSH, NMSE-AOSH) and the original method (NME-AOSH) in different ${\Delta _{s}}$ are listed in Tables 2 and 3.

**Table 2:** Compression performance of the reconstructed image of the dice.

${\Delta _{s}}$	Method	Lines	PSNR (dB)	R (%)
2	NME-AOSH (original)	793	39	–
	NRMSE-AOSH	674	38	15
	NMSE-AOSH	673	38	15
3	NME-AOSH (original)	636	38	–
	NRMSE-AOSH	515	37	19
	NMSE-AOSH	513	37	19
4	NME-AOSH (original)	530	38	–
	NRMSE-AOSH	414	38	22
	NMSE-AOSH	411	38	22
5	NME-AOSH (original)	433	38	–
	NRMSE-AOSH	350	37	21
	NMSE-AOSH	343	37	23
6	NME-AOSH (original)	373	37	–
	NRMSE-AOSH	306	37	18
	NMSE-AOSH	294	37	21
7	NME-AOSH (original)	327	37	–
	NRMSE-AOSH	273	37	17
	NMSE-AOSH	257	36	21

The symbol “–” means that the original method does not compare with itself.

**Table 3:** Compression performance of the reconstructed image of the United States Air Force resolution chart.

${\Delta _{s}}$	Method	Lines	PSNR (dB)	R (%)
2	NME-AOSH (original)	1242	35	–
	NRMSE-AOSH	853	34	31
	NMSE-AOSH	621	33	50
3	NME-AOSH (original)	1017	34	–
	NRMSE-AOSH	625	33	39
	NMSE-AOSH	470	32	54
4	NME-AOSH (original)	803	32	–
	NRMSE-AOSH	498	31	38
	NMSE-AOSH	388	31	52
5	NME-AOSH (original)	730	32	–
	NRMSE-AOSH	423	31	42
	NMSE-AOSH	323	30	56
6	NME-AOSH (original)	625	32	–
	NRMSE-AOSH	368	31	41
	NMSE-AOSH	283	31	55
7	NME-AOSH (original)	576	32	–
	NRMSE-AOSH	326	30	43
	NMSE-AOSH	249	30	57

The symbol “–” means that the original method does not compare with itself.

According to the results in Tables 2 and 3, we conclude that the new method of AOSH (NRMSE-AOSH, NMSE-AOSH) have a better compression performance compared to the original method of AOSH (NME-AOSH) since the number of scanned lines required by the new method for AOSH is less than that of the original method (where *R* is 15–23% for the dice and 38-56% for the United States Air Force (USAF) resolution chart). This is not surprising because the USAF chart tends to have more similarity than the dice because it is made up of regular groups of horizontal strips and vertical strips. Therefore, *R* is object-dependent. We also note that *R* corresponds to the reduction of time by the same rate, assuming the time to acquire successive y-scan is the same, which is true in practice. The degradation of the reconstructed image of the two new methods, however, is extremely close to that of the original method. Also, as we previously mentioned, the factor $\Delta _{s}$ in the AOSH system decides the quality of the reconstructed image represented by the scanned lines.

For a given object and method, a larger $\Delta _{s}$ corresponds to a reduced number of scanned lines in AOSH. The rate *R* exhibits slight variations when modifying $\Delta _{s}$ within the same AOSH method, indicating the stability and validity of the proposed new AOSH techniques. Moreover, it is noteworthy to note that the proposed two methods display markedly distinct *R* values when applied to two different types of objects (reflective and transmissive), providing further evidence of the adaptability of the new methods in selectively capturing pertinent information.

## Discussion

We have presented two enhanced methods for AOSH technology, which we refer to as NRMSE-AOSH and NMSE-AOSH. The major difference between the new methods (NRMSE-AOSH, NMSE-AOSH) and the original AOSH (NME-AOSH) is that the two of new methods use a better error evaluation method in model evaluation instead of using NME. Theoretically, both NRMSE and NMSE could better show the similarity between hologram lines, thereby improving the compression performance of AOSH. As such, both the overall time required to scan the scene and the storage space needed are reduced in NRMSE-AOSH and NMSE-AOSH. The improvement in the hologram acquisition process is extremely important for wide-field applications, in which case lengthy capturing time in the original AOSH method is needed. We have evaluated our proposed new methods by capturing holograms of two different types (transmissive and reflective) of objects. Comparing to the original AOSH method (NME-AOSH), the new methods of AOSH shown better performance on the reduction of scanned lines, as shown in Tables 2 and 3. The results show that NRMSE-AOSH and NMSE-AOSH are faster than the original AOSH up to $R=50\%$, while preserving favorable quality on the reconstructed images. Besides, NMSE-AOSH shows the best compression performance for the objects among these methods because of the mathematical properties of NMSE. Consequently, we firmly believe that the advantageous attributes of the NMSE-AOSH method will usher in notable advancements in the domain of large-scale dynamic hologram acquisition, particularly in scenarios necessitating substantial expansion of the hologram size to faithfully represent wide-field scenes.

## Data Availability

All data generated or analysed during the current study are included in this published article.
